# High Fasting Insulin Levels and Insulin Resistance May Be Linked to Idiopathic Recurrent Pregnancy Loss: A Case-Control Study

**DOI:** 10.1155/2013/576926

**Published:** 2013-11-25

**Authors:** Corina-Alina Ispasoiu, Radu Chicea, Florin Vasile Stamatian, Florin Ispasoiu

**Affiliations:** ^1^Astra Clinic, Gr. Alexandrescu Street, No. 1, 550371 Sibiu, Romania; ^2^Obstetrics and Gynaecology Department, University of Medicine “Victor Papilian”, L. Blaga Street, No. 2-4, 550169 Sibiu, Romania; ^3^1st Obstetrics and Gynaecology Department, University of Medicine and Pharmacy, “Iuliu Hatieganu”, Clinicilor Street, No. 1-3, 400006 Cluj-Napoca, Romania; ^4^Polisano Clinic, Constitutiei Street, No. 24, 550253 Sibiu, Romania

## Abstract

*Objective*. Patients with more than two spontaneous pregnancy losses are diagnosed with recurrent pregnancy loss. The aim of this study was to evaluate the IR (insulin resistance) in patients with idiopathic recurrent pregnancy loss. *Material and Method*. A single center, case control study was performed on one hundred eighteen women divided into case group (with at least two pregnancy losses, earlier than 20 weeks of gestation, and negative for the recurrent pregnancy loss testing) and control group (with at least one live birth, no pregnancy loss). FG (fasting glucose) and FI (fasting insulin) were determined for all patients. IR was evaluated by HOMA-IR index. *Results*. There were not significant differences between the mean age and BMI in cases and controls (*P* > 0.05). Fasting glucose was significantly higher in the control group (85.6 versus 79.8 *P* < 0.01), but fasting insulin (15.24 versus 12.83, *P* < 0.001) and HOMA-IR (2.98 versus 2.69, *P* < 0.05) were significantly higher in the case group. *Conclusion*. In women with idiopathic recurrent pregnancy loss FI and IR are higher than those in women without spontaneous abortion.

## 1. Introduction

Conventionally, the recurrent pregnancy loss was defined as three consecutive losses earlier than 20 weeks of gestation, but testing the women after 2 losses could spare them of another pregnancy failure; thus the definition was modified lowering the number of spontaneous losses to two [1]. 

Recurrent pregnancy loss affects 2%–4% of reproductive-age couples [2], representing a challenge for the physicians, affecting both naturally conceived pregnancies and those obtained after assisted reproductive technology treatment [3]. 

In the etiology of RPL a broad spectrum of factors has been described: chromosome anomalies, uterine malformations or anomalies, immunological factors, hypothyroidism, cervical incompetence, antiphospholipid syndrome, bacterial infections, and polycystic ovary syndrome (PCOS) [4] but half of the cases remain unexplained. 

PCOS is the most common endocrine disorder in women, with prevalence between 6% and 15% (when the broader Rotterdam criteria are applied) [5]. The mechanisms through which pregnancy loss occurs in patients with PCOS include obesity, hyperinsulinemia, IR (insulin resistance), hyperandrogenemia, poor endometrial receptivity, and elevated levels of LH [6]. Insulin resistance and hyperinsulinemia are claimed to be a potential cause of the high rate of pregnancy loss in patients with PCOS and have been linked to the metabolic and endocrine abnormalities associated with the physiopathology of recurrent pregnancy loss. Using the fasting blood glucose, fasting insulin, and HOMA (homeostasis model assessment) score, the insulin resistance was found three times higher in an unselected population of women with recurrent pregnancy loss when compared with normal population [7]. 

Several studies demonstrated that the use of metformin in the treatment of PCOS reduces the risk of spontaneous abortion [8] by decreasing the IR. It was therefore concluded that the IR is the key link between PCOS, obesity, and the recurrent pregnancy loss.

This study aims to evaluate the relation between IR and the recurrent pregnancy loss in a group of patients whom diagnosis was idiopathic recurrent pregnancy loss. 

## 2. Materials and Method

This was a single center, case-control study, conducted at the Obstetrics and Fertility Department of Polisano Clinic, Sibiu, Romania. All patients with 2 or more pregnancy losses addressing to our center between January 2011 and December 2012 were eligible to participate in the study. All the pregnancy losses were documented by ultrasound or histological exam after uterine curettage. 

All the women underwent the following exams: hysterosalpingography, karyotypes of both partners, serum TSH, FT4, prolactin, antibodies for antibeta2-GPI, anticardiolipin, lupus anticoagulant, study of genetic thrombophilic mutations (factor V Leiden; prothrombin; G20210A and methylenetetrahydrofolate reductase MTHFR C677T, A1298C; PAI-1; Antithrombin III), and proteins C and S.

All patients with none of the above factors were included in the case group. The control group consisted in women with no pregnancy loss, with at least one live birth. The exclusion criteria for both groups were the presence of diabetes mellitus (the fasting glucose level higher than 126 mg/dL, at two different testing levels) or the presence of PCOS. PCOS status was determined based on the revised Rotterdam criteria: (1) oligo- and/or anovulation, (2) clinical and/or biochemical signs of hyperandrogenism, and (3) polycystic ovaries with exclusion of other etiologies (congenital adrenal hyperplasia, androgen-secreting tumors, or Cushing's syndrome). The diagnosis was made if any 2 out of 3 criteria were met [8].

The ethical approval for the study was obtained from the institutional reviewer board and the consent was obtained from patients. 

All patients in both groups were tested for fasting glucose (FG) and fasting insulin levels (FI). Based on these tests, the HOMA-IR (homeostasis model assessment of insulin resistance) index was calculated using the formula:

(1) $$\begin{array}{c} \text{HOMA-IR}=\;\frac{\text{FG}\left(\text{mg}/\text{dL}\right)\times \text{FI}\left(\mu \text{U}/\text{mL}\right)}{405}. \end{array}$$

The patients were asked to fast 12 hours before the blood test. The venous blood was analyzed using glucose oxide method and ECL method for insulin levels. 

The primary outcome measure was HOMA-IR levels in both groups. The secondary outcome measures were the FG and FI. Additional analysis was performed on age and BMI. 

The statistical analysis was performed with IBM SPSS software, version 18. Age, BMI, FG, and FI levels in both groups were compared using independent samples *t*-test; chi-square test was used for the determination of significance between groups. The statistical significance was accepted when *P* ≤ 0.05.

## 3. Results and Discussion

121 patients with RPL who are addressed to our department were eligible to participate in the study. After completing all the above-mentioned tests, 71 patients were included in the case group. In the control group we included 60 patients. After testing for diabetes mellitus and PCOS, 6 patients in case group and 7 patients in the control group were excluded. Finally, 65 patients in case group and 53 patients in control group were analyzed.

**Figure 1:**
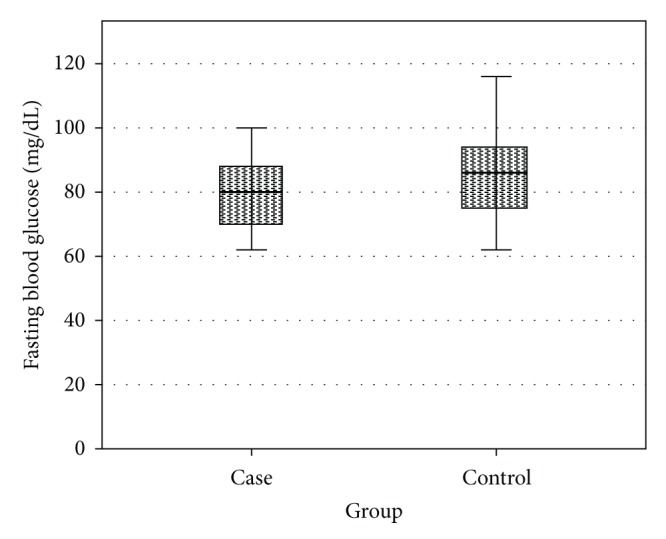
Comparison of fasting glucose levels in case and control groups.

**Figure 2:**
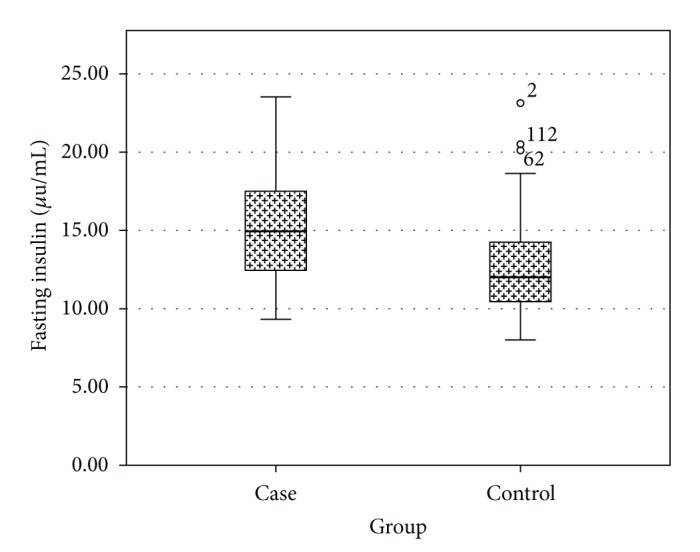
Comparison of fasting insulin levels in case and control groups.

**Figure 3:**
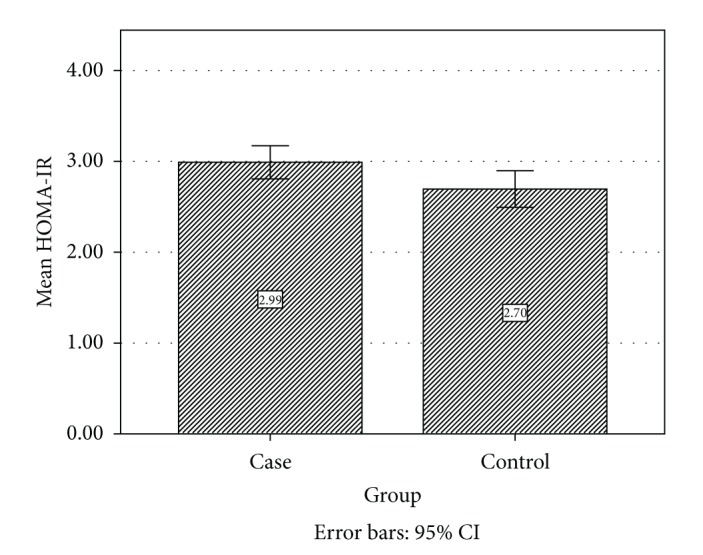
Comparison of HOMA-IR in case and control groups.

As shown in Table 1, no significant differences were observed between the two groups in terms of mean age and mean BMI. Fasting glucose was significantly higher in the control group compared to case group (79.8 ± 10.1 mg/dL versus 85.6 ± 13.1 mg/dL, *P* < 0.01) (Figure 1); fasting insulin was significantly higher in the case group (15.24 ± 3.5 *μ*U/mL versus 12.83 ± 3.2 *μ*U/mL, *P* < 0.001) (Figure 2); also HOMA-IR was significantly higher in the case group (2.98 versus 1.69, *P* < 0.05) (Figure 3). 

The results of our study support the idea that IR may be involved in the etiology of recurrent pregnancy loss. In our study, the FI and the HOMA-IR values were significantly higher in the case group, suggesting that not only the IR, but also the hyperinsulinemia may have an adverse effect on the outcome of pregnancy. 

The studies which focused on recurrent pregnancy loss propose different etiologies: genetic factors (2–5%); anatomic factors (10–15%); endocrine diseases (17–20%): PCOS, uncontrolled diabetes mellitus, thyroid diseases, and hyperprolactinemia; infectious factors (0.5–5%); immunologic factors (8–42%): antiphospholipid syndrome, autoimmune diseases; and thrombotic factors. There is also some supportive scientific evidence for environmental, occupational, and male factors [9]. However, when all known and potential causes for recurrent pregnancy loss are accounted for, almost half of patients will remain without a diagnosis.

The elevated blood insulin and the increased IR create a status similar to that of patients with PCOS, and there are several studies suggesting that hyperinsulinemia and IR are associated with recurrent pregnancy loss [10, 11].

Patients with diabetes and PCOS were excluded from the study and the BMI was similar between the two groups, so the elevation of IR may not be attributed to these two conditions known for increasing it. 

The association between recurrent pregnancy loss and IR is difficult to explain. Insulin resistance is often associated with a hypercoagulable state (impaired fibrinolysis) and increased inflammatory cytokine levels [12]. Insulin resistance has been demonstrated to increased expression of PAI-1. PAI-1 activity is known to elevate levels of serum insulin and it induces a hypofibrinolytic state. This creates a thrombotic milieu at the maternofetal interface with high risk of miscarriage [11]. Insulin resistance is known to play a critical role in the ovarian androgen excess and therefore might promote miscarriage by increasing circulating testosterone concentrations. 

The expression of glycodelin and of IGFBP1 (insulin-like growth factor-binding protein 1) is decreased by the hyperinsulinemia at the implantation situs [13]. Glycodelin plays an immune role, inhibiting the endometrial response towards the embryo, while the IGFBP1 facilitates the adhesion process of the blastocyst at the fetomaternal interface [14].

There are studies suggesting that hyperinsulinaemia and/or the IR syndromes may have various deleterious metabolic effects, including causing an increase of plasma hyperhomocysteinemia [15]. Elevated homocysteinemia may impair pregnancy by interfering with endometrial blood flow and vascular integrity [16]; it increases the oxidative stress in vascular endothelium, activates the platelets, and has been documented to increase the probability of early pregnancy loss [17]. 

The PCOS-like status, due to elevated FI and high IR, and the successful use of metformin for the treatment of PCO suggested the idea that metformin could be used in these women. The beneficial effects of metformin use are a decrease in the level of PAI1 (plasminogen activator inhibitor-1) [18], enhancement of the uterine vascularity [19, 20], and alleviation of the IR, insulin levels, and HOMA-IR. Insulin resistance has further been associated with abnormal endometrial development and endometrial defects. One study reported that lowering the insulin levels with metformin enhances uterine vascularity and reduces uterine vascular resistance; after metformin use the vascular resistance in spiral arteries was reduced with 20% (0.71 ± 0.02 to 0.57 ± 0.03  (*P* < 0.001)) [19]. 

Our study is to our knowledge the only one including Romanian patients with idiopathic recurrent pregnancy loss. Our study used a small sample size and by using the HOMA-IR as an indicator of IR may not be as accurate as the euglycemic clamp but is a cheaper method, less invasive, easy repeatable, and applicable for larger groups of patients. 

## 4. Conclusion

Current study showed that, in women with idiopathic recurrent pregnancy loss, FI and IR are higher than in women without abortion but further more comprehensive studies are needed in the future. We recommend that fasting glucose and fasting insulin levels should be measured in all women with recurrent pregnancy loss.

## Figures and Tables

**Table 1:** Comparison of age and BMI in case and control groups.

	Group	*N*	Mean	Std. deviation	Std. error mean
Age	Case	65	30.12	4.904	0.608
	Control	53	29.36	5.274	0.724

BMI	Case	65	26.2283	4.71801	0.58520
	Control	53	25.6653	4.87006	0.66895

## Abbreviations

IR: Insulin resistance
FI: Fasting insulin
FG: Fasting glucose
HOMA-IR: Homeostasis model of assessment-insulin resistance
BMI: Body mass index
PCOS: Polycystic ovary syndrome
IGFBP1: Insulin-like growth factor binding protein 1
PAI1: Plasminogen activator inhibitor 1
ECL: Electrochemiluminescence.
